# Case Report: A rare case of intestinal and mucinous-type renal pelvis adenocarcinoma masked by complex renal calculi: a diagnostic dilemma and therapeutic challenge

**DOI:** 10.3389/fonc.2026.1698768

**Published:** 2026-01-21

**Authors:** Yuning Xie, Yuchen Sun, Xianzhen Yang, Xuanyan Che, Fengyue Li, Yifei Wang, Xiande Cao

**Affiliations:** Affiliated Hospital of Shandong University of Traditional Chinese Medicine, Jinan, Shandong, China

**Keywords:** chronic inflammation, intestinal type adenocarcinoma, mucinous adenocarcinoma, renal pelvis adenocarcinoma, urolithiasis

## Abstract

Primary renal pelvis adenocarcinoma (RPA), particularly the mucinous subtype, is an exceedingly rare and aggressive malignancy often associated with chronic inflammation and long-standing calculi, making its diagnosis challenging due to non-specific symptoms mimicking common urological conditions. We present a 79-year-old male patient with a decade-long history of complex nephrolithiasis and recurrent infections, who underwent multiple percutaneous nephrolithotomy (PCNL) procedures. A pivotal diagnostic moment arose during a subsequent PCNL when extensive “purulent moss” was found without significant residual stone fragments, prompting biopsy. Histopathology confirmed high-grade mucinous and intestinal-type adenocarcinoma of the renal pelvis, subsequently managed with laparoscopic radical nephroureterectomy. This case underscores the diagnostic challenge posed by RPA, often masked by chronic calculous disease, emphasizing the critical importance of a high index of suspicion and prompt histopathological evaluation of atypical intraoperative findings (e.g., “purulent moss” instead of expected stone) in patients with complicated urolithiasis, facilitating early diagnosis and improving outcomes.

## Introduction

Primary renal pelvis adenocarcinoma (RPA) is a rare and aggressive malignancy, constituting a minute fraction of all renal and urinary tract neoplasms (1–3). While urothelial carcinoma is the predominant histological subtype in the renal pelvis, adenocarcinomas are far less common. Among these rare adenocarcinomas, the mucinous type is exceptionally infrequent (4), often arising from glandular metaplasia within the renal pelvic urothelium, a change frequently induced by chronic irritation, infection, or long-standing calculi (5).

The insidious nature of RPA, coupled with its non-specific clinical manifestations, presents significant diagnostic challenges. Patients commonly experience symptoms mimicking more prevalent benign urological conditions, such as nephrolithiasis, urinary tract infections, or hydronephrosis, thereby masking the underlying malignant process and often leading to delayed diagnosis (6, 7). Chronic calculous disease and recurrent pyelonephritis are well-established risk factors for RPA, creating a persistent inflammatory milieu that can predispose the urothelium to malignant transformation (8, 9).

Herein, we report a highly compelling case of primary mucinous adenocarcinoma of the renal pelvis in an elderly male patient with a decade-long history of complex renal calculi and recurrent urinary tract infections. This case uniquely illustrates the profound diagnostic dilemma encountered when such a rare malignancy is obscured by long-standing, symptomatic calculous disease. Notably, the tumor was ultimately diagnosed following multiple percutaneous nephrolithotomy (PCNL) procedures, where atypical tissue, rather than the expected stone, prompted histological examination. This report aims to underscore the critical importance of heightened clinical suspicion, diligent intraoperative assessment, and prompt histopathological evaluation in patients presenting with refractory or atypical renal calculous disease, particularly in the context of chronic inflammation.

## Case presentation

A 79-year-old male patient with a prolonged history (over 10 years) of untreated nephrolithiasis initially presented at a local hospital on 9 January 2025, complaining of left flank and abdominal pain of an unspecified duration. At this initial presentation, he denied systemic symptoms such as fever, urinary frequency, urgency, or dysuria.

Initial computed tomography (CT) on 9 January 2025 revealed significant left hydronephrosis and ureterectasis, with multiple calculi identified in the left renal pelvis, calyces, and proximal ureter. Laboratory investigations on 10 January 2025 showed a mild leukocytosis (WBC count 11.47 × 10^9^/L) and elevated urine white and red blood cell counts, while serum creatinine was 92.8 µmol/L (GFR 67.2 mL/min). Given the clear evidence of obstruction and potential infection, an ultrasound-guided percutaneous left pyelostomy and drainage were performed on 10 January 2025. A renal scintigraphy (ECT) on 13 January 2025 confirmed severely impaired left renal function (GFR 12.41 mL/min) with no excretion, alongside slightly reduced right renal function (GFR 37.36 mL/min), resulting in a total GFR of 49.78 mL/min. He was discharged with a plan for definitive surgical stone removal 1 month later. At this stage, malignancy was not suspected, and the focus remained on managing the chronic lithiasis and associated obstruction/infection.

The patient experienced recurrent infective episodes, leading to readmissions. On 30 January 2025, he was re-admitted with acute urinary pain, hematuria, and high-grade fever (39.7°C). Admission CT confirmed left renal calculi, hydronephrosis, and perirenal exudate consistent with pyelonephritis. Markedly elevated inflammatory markers (WBC 27.72 × 10^9^/L, CRP 21.8 mg/L, and PCT 1.593 ng/mL) further supported an acute infection. He responded to intravenous antibiotics and was discharged on 6 February 2025, with a plan for definitive surgery in 3 weeks.

On 25 February 2025, he was admitted for planned stone removal. A repeat ECT showed improved left renal function (GFR 24.27 mL/min) with delayed excretion, likely due to continuous drainage. On 28 February 2025, he underwent PCNL combined with ureteroscopy (URS) and laser lithotripsy. Intraoperatively, multiple calculi were noted, along with extensive “purulent moss”. Because of the substantial stone burden, prolonged surgical time, and severe intra-renal infection, the procedure was terminated, and a two-stage approach was planned, leaving an F14 nephrostomy tube and an F6 ureteral stent *in situ*.

Subsequent readmissions on 24 March 2025 and 2 April 2025 for second- and third-stage PCNLs continued to reveal similar findings. During these procedures, multiple stones were found accompanied by adherent “purulent moss” and severe adhesion to the renal calyx wall. Despite extensive laser lithotripsy and stone removal, significant “purulent moss” persisted. While the collecting system appeared free of other calculi after the third procedure, the persistent presence of this unusual material, initially attributed solely to chronic infection and reactive changes, obscured the underlying pathology.

The diagnostic trajectory took a critical turn on 3 June 2025. A follow-up CT scan at a local hospital, while noting persistent stones, hydronephrosis, and perirenal exudate, for the first time suggested to “rule out ureteral tumor”. This marked the first imaging-based suspicion of a neoplastic process.

On 8 June 2025, the patient was initially presented to our hospital again due to persistent infection; non-contrast KUB CT was first performed at our institution (shown in **Supplementary Figures S1–S26**). A PCNL and ureteral stent insertion were performed on 12 June 2025. Intriguingly, during this procedure, despite prior CT findings suggesting residual stones, a large amount of “purulent moss”—tumor tissue (high-grade mucinous and intestinal-type adenocarcinoma) mimicking inflammatory exudate—was encountered, but no significant residual stone fragments were found after thorough exploration of the renal collecting system. This discrepancy between imaging findings, chronic infection, and the atypical intraoperative findings of tenacious “purulent moss” in the absence of significant stones prompted suspicion of an underlying malignancy. Consequently, a renal pelvis tissue sample was collected for pathological examination.

**Figure 1:**
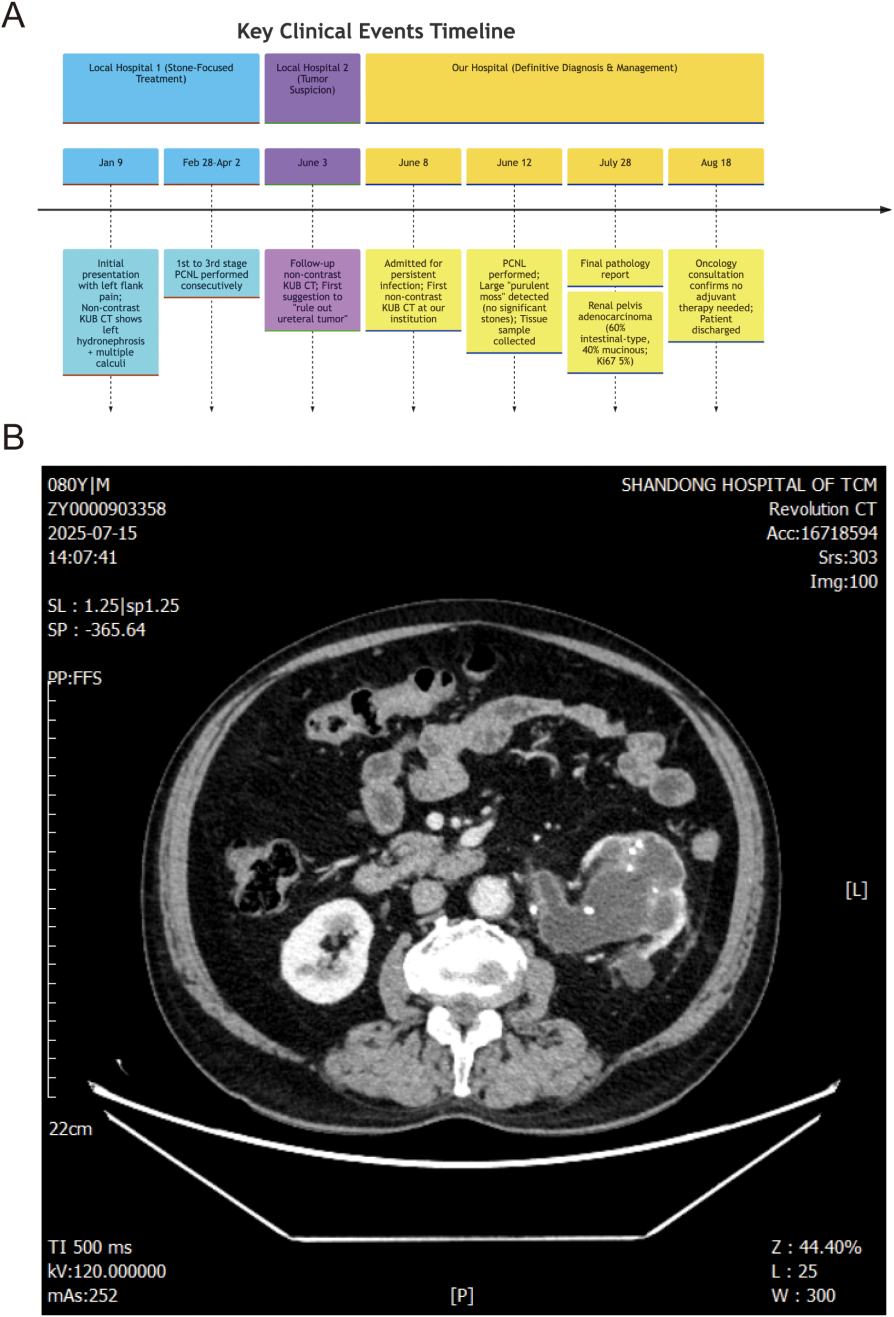
**(A)** Patient diagnosis and treatment route map. **(B)** Abdominal CT image demonstrating an enlarged kidney accompanied by multiple calculi.

The pathology report on 17 June 2025 provided the definitive diagnosis: high-grade tubular adenocarcinoma of the left renal pelvis tissue. Immunohistochemical (IHC) staining revealed positivity for CK7 (cytoplasmic), CK20 (cytoplasmic), CDX-2 (nuclear), and SATB2 (nuclear), weak P504S (cytoplasmic) and PAX-8 (nuclear), and membrane Beta-catenin, with a high Ki67 proliferation index (nuclear, 70%), strongly supporting an adenocarcinoma with intestinal differentiation. This confirmed that the “purulent moss” was, in fact, tumor tissue mimicking inflammatory exudate (**Figure 1A**).

**Figure 2:**
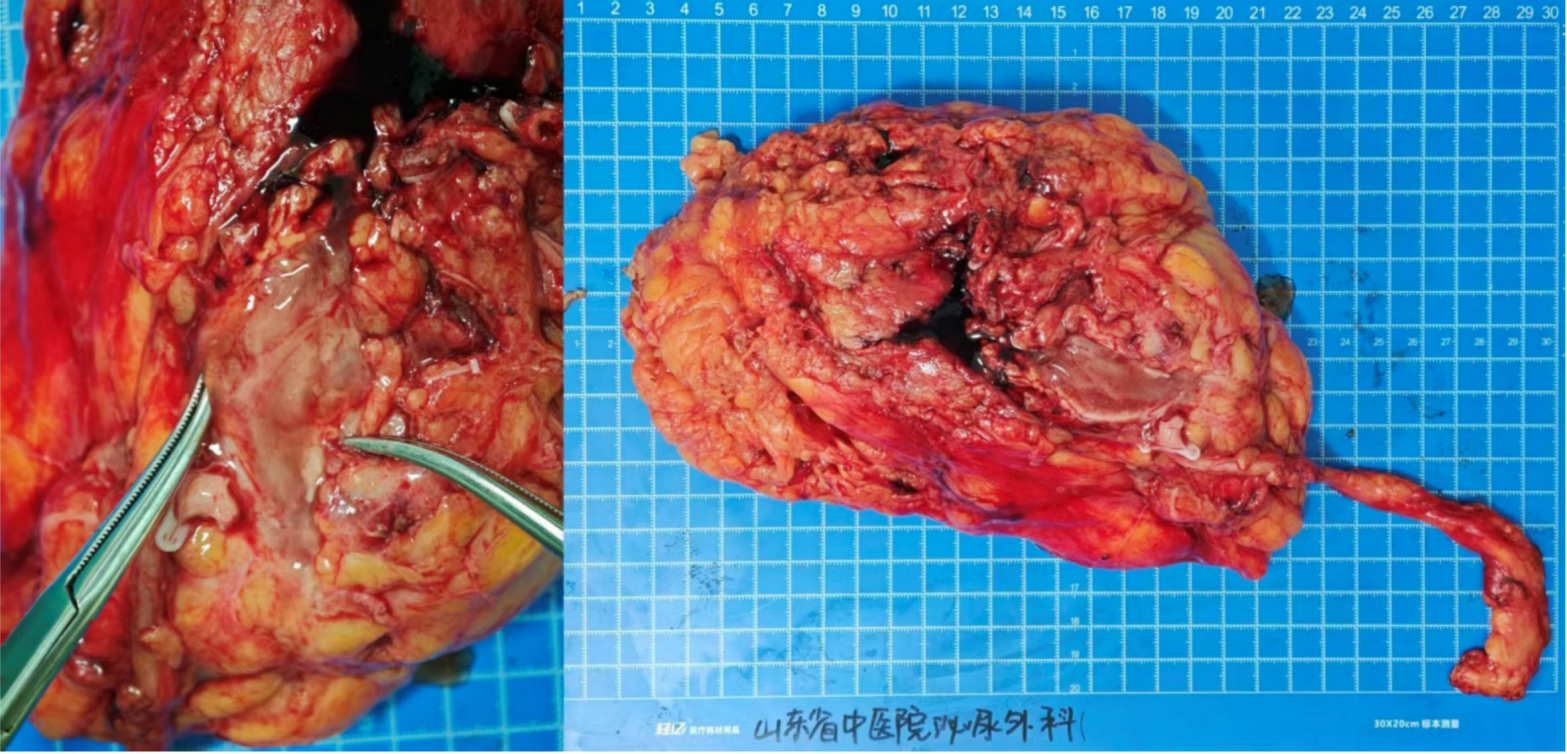
Schematic diagram of total nephrectomy. The left panel depicts the incisional site with mucinous exudate. The right panel provides a panoramic view of the removed kidney.

Following this definitive diagnosis, the patient was admitted for definitive surgical management on 14 July 2025. A CT scan on 15 July 2025 specifically identified an “occupying lesion” in the left kidney and renal pelvis (**Figure 1B**), recommending further pathological assessment. To exclude a primary gastrointestinal source for the adenocarcinoma, an electronic gastroscopy and colonoscopy were performed on 17 July 2025, which showed no abnormalities in the colon and only esophagitis on pathology. With the absence of an identifiable primary tumor elsewhere, a laparoscopic left radical nephroureterectomy was performed on 22 July 2025 (**Figure 2**).

**Figure 3:**
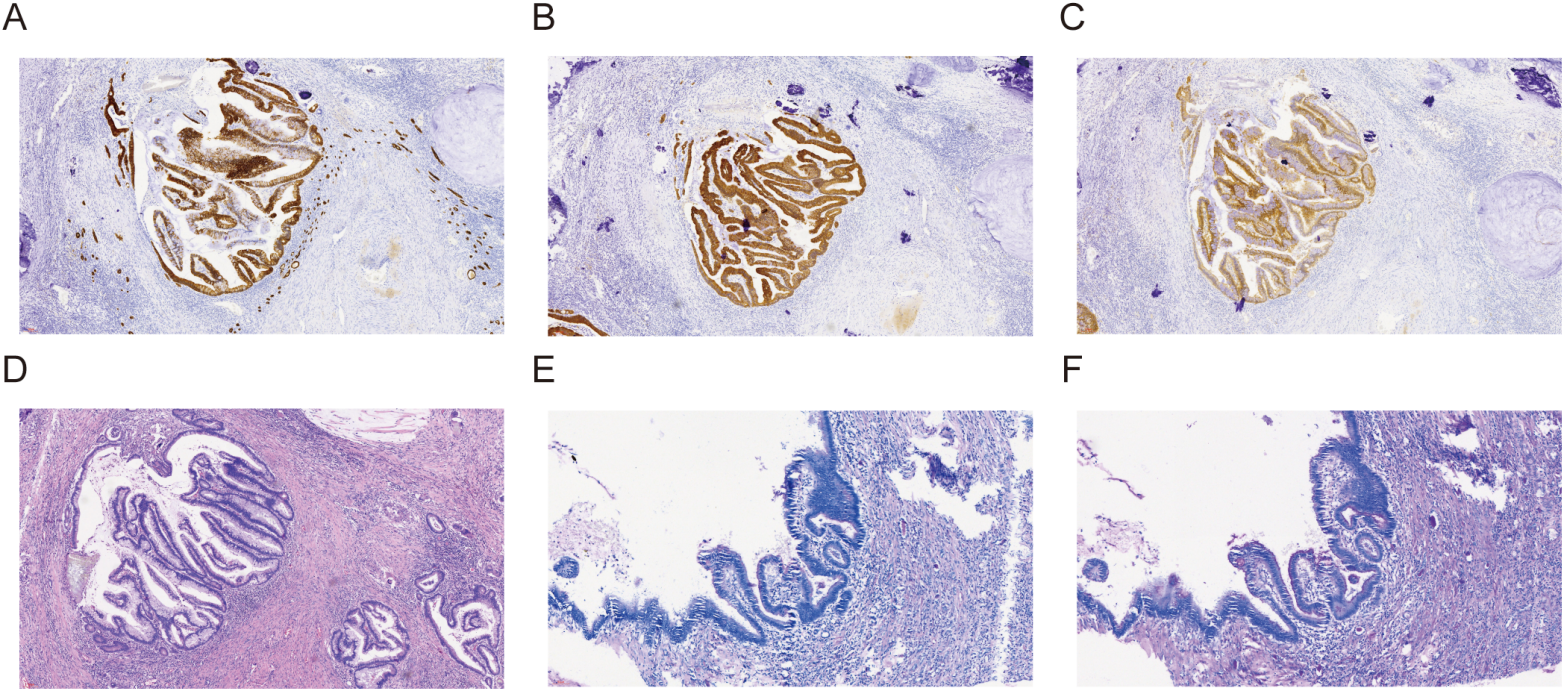
Immunohistochemical staining of the renal pelvic mucinous adenocarcinoma. The tumor cells showed strong positive expression for **(A)** CK7, **(B)** CK20, and **(C)** Villin, indicating an intestinal-type differentiation. **(D)** HE staining revealed an infiltrative adenocarcinoma composed of glandular structures lined by columnar and goblet cells, with abundant extracellular mucin pools. **(E)** PAS staining demonstrated strong positivity for mucin within the glandular lumens and extracellular mucin pools, which appeared bright magenta. **(F)** D-PAS staining remained positive after diastase digestion, confirming the presence of neutral mucin.

The final comprehensive pathology report on 28 July 2025 confirmed RPA, composed of 60% intestinal-type and 40% mucinous adenocarcinoma components. The tumor had invaded the renal sinus fat but did not show vascular tumor emboli, involvement of ureteral or vascular margins, or perirenal fat invasion. The extensive IHC profile from the resected specimen [CK7 (+), CK20 (+), Villin (apical cytoplasmic+), SATB2 (+), PAX-8 (−), Ki67 (5%), cytoplasmic Beta-catenin+, CD31 (membranous+), D2-40 (lymphatics+), and S-100 (cytoplasmic−)] further solidified the diagnosis of primary renal pelvic mucinous adenocarcinoma with intestinal differentiation (**Figure 3**).

The patient’s post-operative recovery was stable, with a GFR of 66.14 mL/min on 31 July 2025. An oncology consultation on 18 August 2025 concluded that no evidence of lymph node metastasis or perirenal fat invasion was present, and therefore, no immediate adjuvant therapy was indicated. The patient was discharged in stable condition.

## Discussion

This case vividly exemplifies the profound diagnostic dilemma encountered with primary RPA, particularly when its insidious nature is masked by chronic, symptomatic benign urological conditions. Our patient’s decade-long history of complex nephrolithiasis and recurrent urinary tract infections created a convincing façade for his true pathology. The initial focus on stone clearance and infection management, while clinically appropriate given the presentation, inadvertently delayed the recognition of the underlying malignancy.

Conventional imaging modalities, such as CT, initially proved insufficient for definitive tumor identification amidst the extensive inflammation and stone burden. The limitations of CT in detecting subtle or infiltrative tumors within the context of severe inflammatory and calculous disease are well-documented (10–12). Indeed, the initial CT scans did not suggest a neoplastic process. It was not until much later, after several PCNL procedures, and potentially a subtle evolution in the tumor’s presentation, that a follow-up CT at an external institution cautiously suggested the possibility of a ureteral tumor, highlighting the challenges of imaging in these complex scenarios. The dense calcifications (13, 14), associated hydronephrosis (15, 16), and inflammatory exudates can effectively obscure nascent or even moderately sized neoplastic lesions, leading to a focus on the more obvious calculous disease. It is important to note that the CT scans performed at our institution, including the initial evaluation on 8 June 2025 and subsequent follow-ups, were all non-contrast KUB CTs. This decision was made to avoid potential further damage to the patient’s already compromised renal function from contrast agents.

The true turning point in this patient’s diagnostic journey occurred during the third PCNL. The unexpected finding of extensive “purulent moss”—a soft, grayish-white, irregular, and highly adherent tissue—without significant residual calculous fragments, despite prior imaging suggesting stones, was the critical clue. This atypical observation, diverging from the expected findings during a routine stone removal procedure, shifted the clinical suspicion from mere stone disease and infection to an underlying neoplastic process. This prompted the judicious decision to obtain a tissue biopsy, which ultimately yielded the definitive diagnosis. This highlights a crucial lesson: while “purulent moss” is often associated with chronic infection and struvite stones, its presence without corresponding stone material, or with unusually adherent stones, should trigger a heightened suspicion for malignancy and warrant immediate pathological investigation (17, 18). Histopathology definitively confirmed that this “purulent moss” was, in fact, tumor tissue (high-grade mucinous and intestinal-type adenocarcinoma) mimicking inflammatory exudate, emphasizing the profound diagnostic challenge presented by this rare entity.

The final pathological diagnosis of mucinous and intestinal-type adenocarcinoma is particularly noteworthy. These subtypes are extremely rare within the renal pelvis, representing a distinct entity from the more common urothelial carcinoma (19). Their development is often linked to glandular metaplasia stemming from chronic inflammatory stimuli, such as those caused by long-standing calculi and recurrent infections—a well-established pathomechanism for such malignant transformation, perfectly exemplified by our patient’s history (6, 20). The immune histochemical profile further supported an intestinal differentiation, reinforcing the diagnosis.

A comprehensive differential diagnosis was crucial given the rarity and morphological variability of RPA. The distinctive morphologic features (glandular structures lined by columnar and goblet cells, abundant extracellular mucin pools) and the specific immunophenotype [CK7 (+), CK20 (+), CDX-2 (+), SATB2 (+), Villin (+), PAX-8 (−), and GATA3 (nuclear−)] were instrumental in differentiating primary RPA with intestinal and mucinous differentiation from other potential mimics. Metastatic adenocarcinoma from the gastrointestinal tract was carefully considered and ruled out by extensive endoscopic evaluation (gastroscopy and colonoscopy) showing no primary tumor, alongside the patient’s comprehensive clinical workup and the specific IHC profile. Urothelial carcinoma with glandular differentiation was distinguished by the lack of typical urothelial carcinoma morphology in our case and, crucially, by the strong and diffuse expression of intestinal markers (CDX-2, SATB2, and Villin) along with negative GATA3, which is a common marker for urothelial differentiation. Xanthogranulomatous pyelonephritis (XGP), while capable of mimicking malignancy due to chronic inflammation and mass effect, was ruled out as it is typically characterized by lipid-laden macrophages (foam cells) and granulomatous inflammation, which were absent in both the biopsy and the resected specimen. Renal cell carcinoma (RCC) with mucinous features was excluded due to the PAX-8-negative status in the tumor, as PAX-8 is typically positive in RCCs.

This case offers several crucial clinical implications. Firstly, it underscores the need for an exceptionally high index of suspicion for malignancy in patients presenting with long-standing, complicated urolithiasis and recurrent infections, particularly in older age groups (21, 22). Secondly, clinicians should be acutely aware that atypical intraoperative findings, such as the presence of unusual tissue (e.g., “purulent moss” instead of expected stone material) or disproportionate tissue reaction during endoscopic stone procedures, warrant immediate histopathological evaluation (23, 24). Lastly, persistent or refractory symptoms in the setting of chronic stone disease, even after apparently successful stone clearance, should prompt re-evaluation for alternative underlying pathologies, including rare malignancies.

Regarding the patient’s renal function and the rationale for multiple PCNL procedures: The patient presented with severely impaired left renal function (GFR 12.41 mL/min), which improved to 24.27 mL/min after drainage. Our surgical experts advise that mild to moderate renal function impairment itself is not an absolute contraindication for PCNL; rather, in many cases, it is a significant indication for active surgical intervention to preserve residual function, especially when the impairment is caused by obstruction or infection. The initial PCNLs (first three stages) were performed at an external hospital and aimed to manage the severe, decade-long stone burden and recurrent infections causing obstruction and progressive renal damage. The PCNL at our institution on 12 June 2025 was also primarily intended to alleviate persistent obstruction and infection. This approach is consistent with established clinical practice for stone-related renal dysfunction, where the long-term benefits of relieving obstruction and salvaging renal function often outweigh the short-term risks of surgical intervention. The progressive thinning of the renal cortex observed in later CT scans is understood to be a consequence of this chronic obstruction and recurrent inflammation.

Regarding the management strategy for RPA, owing to its rarity, standard treatment strategies lack high-level evidence and are often guided by principles applied to renal pelvis urothelial carcinoma (25). However, primary RPA is known to be an aggressive malignancy, often diagnosed at an advanced stage with a poor prognosis (26). For localized renal pelvis malignancy, the current recommended standard radical surgical treatment strategy is nephroureterectomy, including excision of the affected kidney, the entire length of the ureter, and a bladder cuff excision (27). This surgical approach aims for complete tumor clearance and to reduce the risk of recurrence in the residual urothelium. Preoperative imaging assessment, particularly CT, is crucial in surgical decision-making. Tumor size and peripelvic fat infiltration on preoperative CT are independent predictors of lymph node metastasis, which aids in preoperative risk stratification and planning the extent of lymphadenectomy (28). In our case, the patient was diagnosed with left RPA based on a biopsy performed during PCNL and a preoperative CT scan showing a left renal pelvis and calyx lesion, with no enlarged retroperitoneal lymph nodes. A laparoscopic left radical nephroureterectomy was performed, and the postoperative pathology confirmed RPA, with 60% intestinal-type and 40% mucinous adenocarcinoma components. Regarding adjuvant therapy, our surgical oncology consultation recommended a combination of chemotherapy and immune checkpoint inhibitors, considering the aggressive nature of the disease. However, the patient and his family declined further adjuvant treatment. The patient is currently under follow-up, and to date, no signs of tumor recurrence or metastasis have been observed. Follow-up includes blood routine, urine routine, liver and kidney function tests, CA199, and thoracoabdominal CT at 1, 3, 6, 9, and 12 months post-surgery. This detailed follow-up strategy reflects a comprehensive post-operative management plan, emphasizing the importance of continued surveillance even in the absence of adjuvant therapy. The standard of care for localized RPA, as performed in this case, remains radical nephroureterectomy (19, 29, 30). The patient’s favorable outcome post-surgery, with no evidence of residual disease, underscores the importance of early diagnosis, despite its inherent challenges.

## Conclusion

Primary mucinous adenocarcinoma of the renal pelvis is an exceedingly rare and aggressive malignancy. This case vividly illustrates its strong association with long-standing renal calculi and recurrent urinary tract infections, which serve as chronic inflammatory drivers. The masquerading symptoms and initial lack of specific imaging findings highlight the considerable diagnostic difficulty, often leading to delays as the disease mimics more common benign urological conditions. Crucially, the diagnosis in our patient was contingent upon recognizing atypical intraoperative findings—specifically the presence of “purulent moss” without significant residual stone during repeat PCNL—prompting a crucial histological examination. Therefore, a high index of suspicion is warranted for patients with chronic, complicated urolithiasis, especially when confronted with unusual or refractory findings during stone management procedures. Timely and thorough histopathological evaluation of suspicious tissues is paramount for early and accurate diagnosis, which is crucial for initiating appropriate definitive treatment and improving patient prognosis.

## Data Availability

The original contributions presented in the study are included in the article/**Supplementary Material**. Further inquiries can be directed to the corresponding authors.
