# Supplementary figures and images for: Case Report: A rare case of intestinal and mucinous-type renal pelvis adenocarcinoma masked by complex renal calculi: a diagnostic dilemma and therapeutic challenge
Source: Front Oncol. 2026 Jan 21;16:1698768. doi: 10.3389/fonc.2026.1698768 (PMC12867860; doi:10.3389/fonc.2026.1698768)

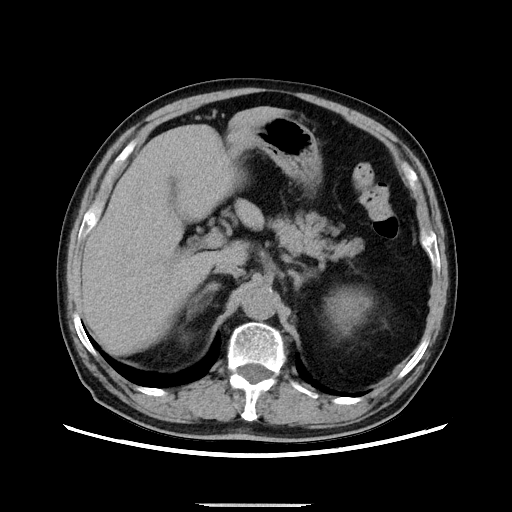

Supplement: Supplementary file 2 [file DataSheet2.zip › Figure S1.tif]

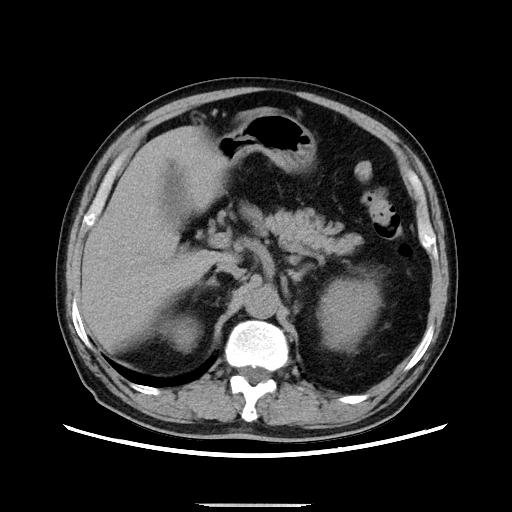

Supplement: Supplementary file 2 [file DataSheet2.zip › Figure S2.tif]

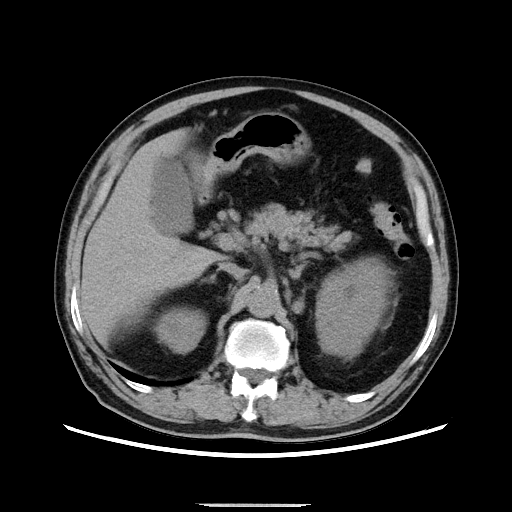

Supplement: Supplementary file 2 [file DataSheet2.zip › Figure S3.tif]

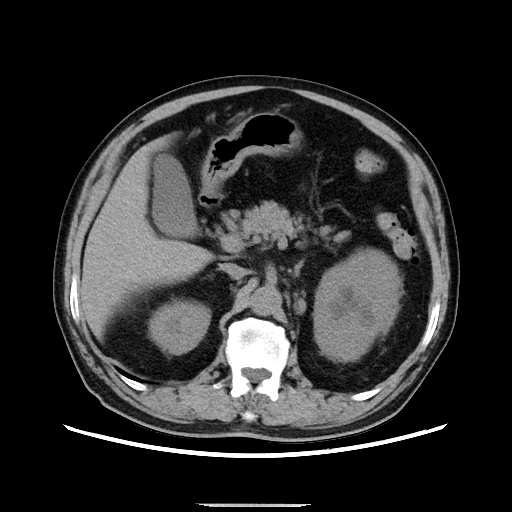

Supplement: Supplementary file 2 [file DataSheet2.zip › Figure S4.tif]

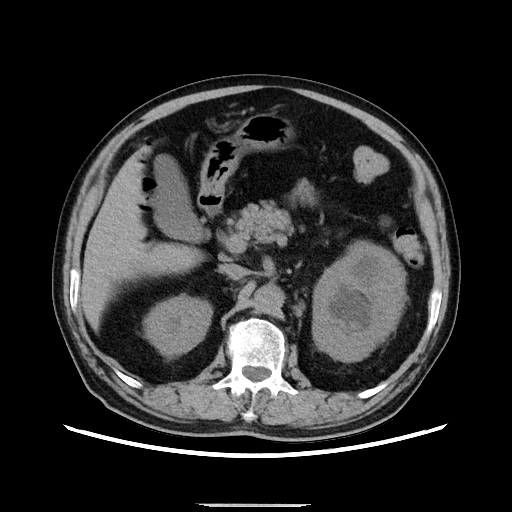

Supplement: Supplementary file 2 [file DataSheet2.zip › Figure S5.tif]

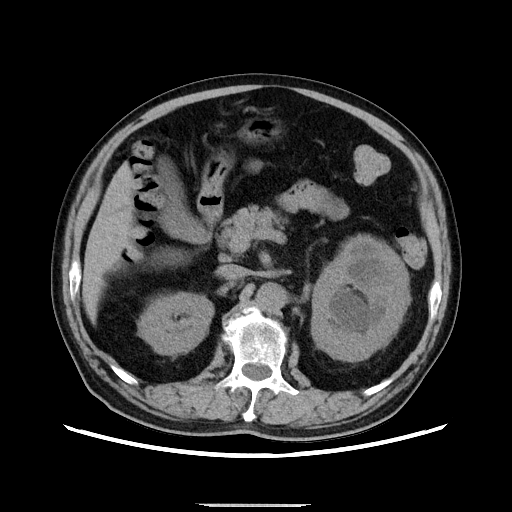

Supplement: Supplementary file 2 [file DataSheet2.zip › Figure S6.tif]

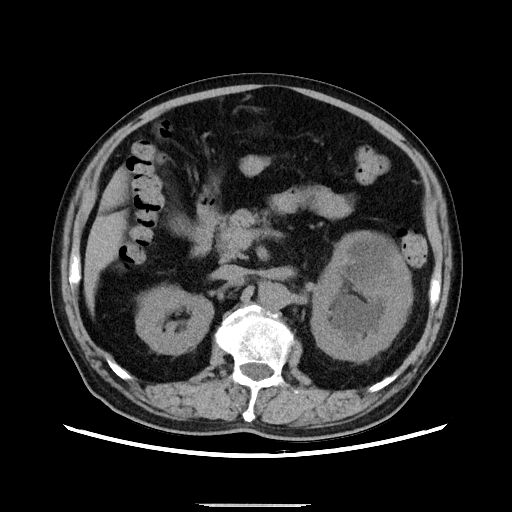

Supplement: Supplementary file 2 [file DataSheet2.zip › Figure S7.tif]

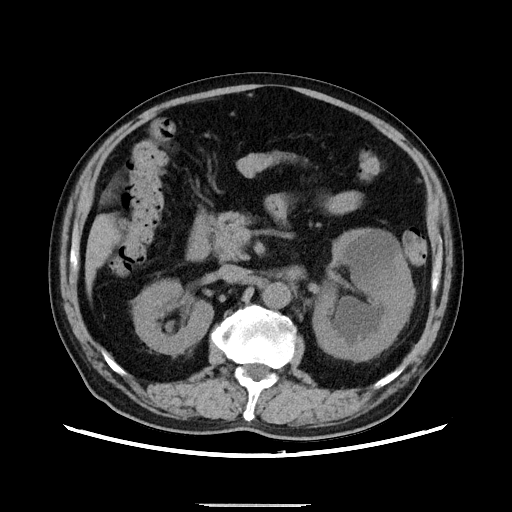

Supplement: Supplementary file 2 [file DataSheet2.zip › Figure S8.tif]

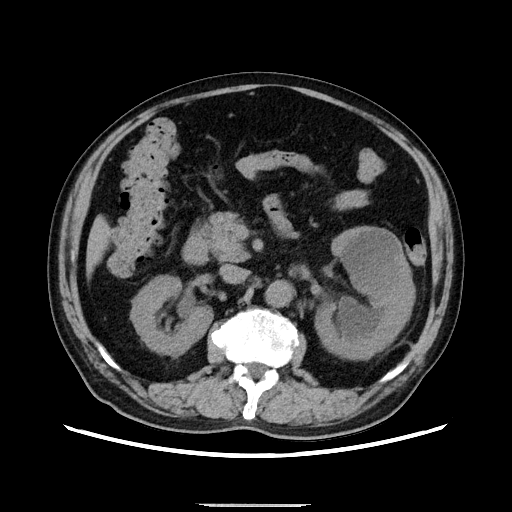

Supplement: Supplementary file 2 [file DataSheet2.zip › Figure S9.tif]

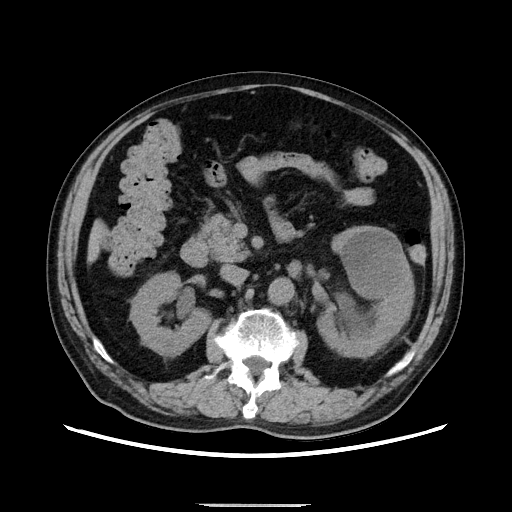

Supplement: Supplementary file 2 [file DataSheet2.zip › Figure S10.tif]

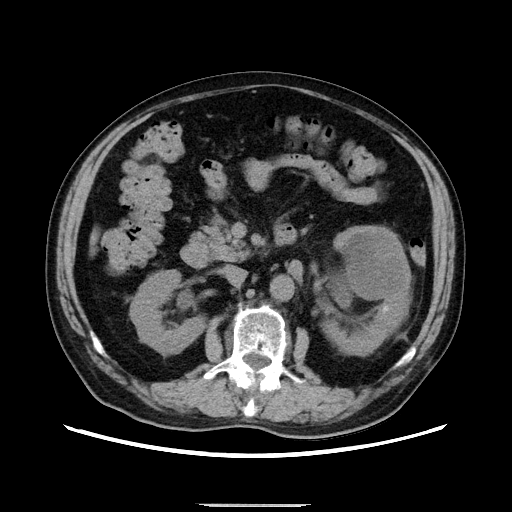

Supplement: Supplementary file 2 [file DataSheet2.zip › Figure S11.tif]

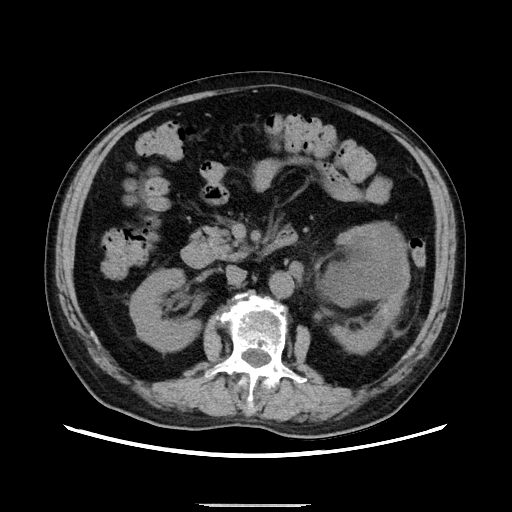

Supplement: Supplementary file 2 [file DataSheet2.zip › Figure S12.tif]

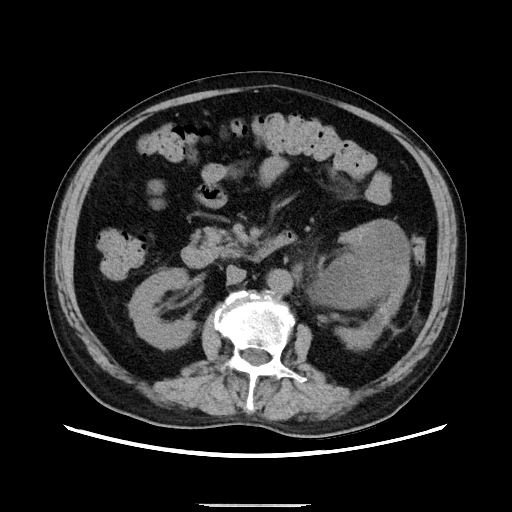

Supplement: Supplementary file 2 [file DataSheet2.zip › Figure S13.tif]

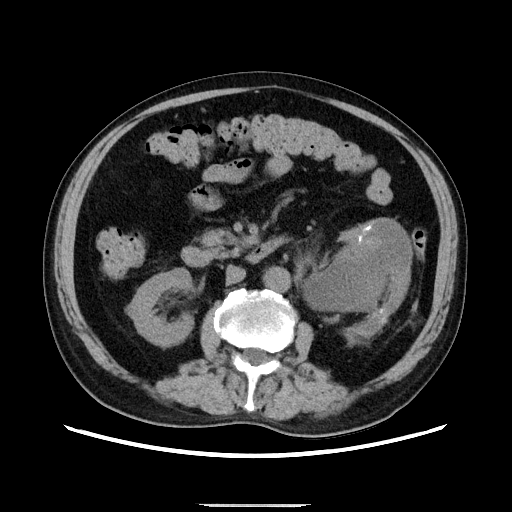

Supplement: Supplementary file 2 [file DataSheet2.zip › Figure S14.tif]

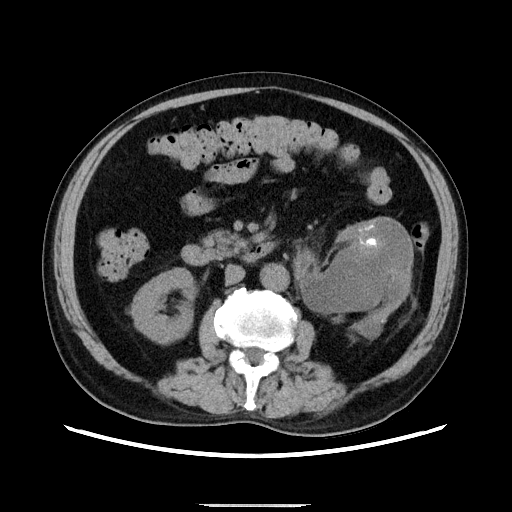

Supplement: Supplementary file 2 [file DataSheet2.zip › Figure S15.tif]

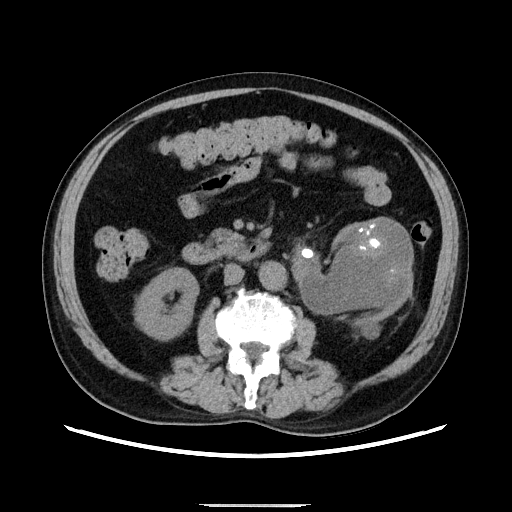

Supplement: Supplementary file 2 [file DataSheet2.zip › Figure S16.tif]

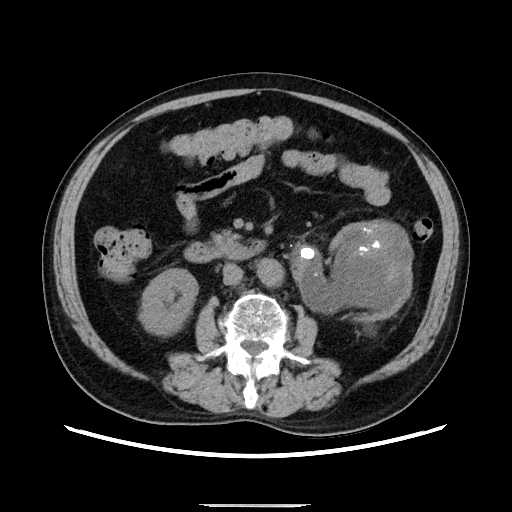

Supplement: Supplementary file 2 [file DataSheet2.zip › Figure S17.tif]

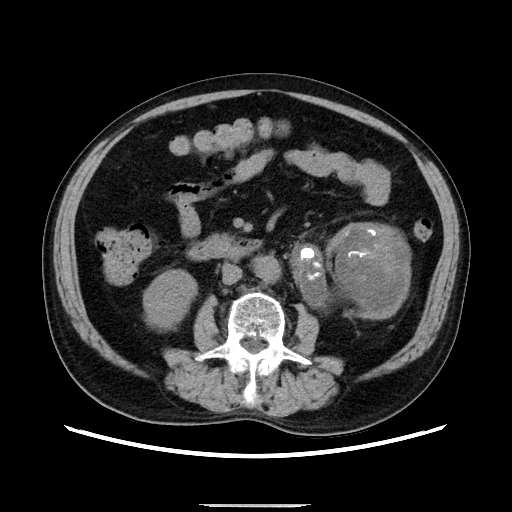

Supplement: Supplementary file 2 [file DataSheet2.zip › Figure S18.tif]

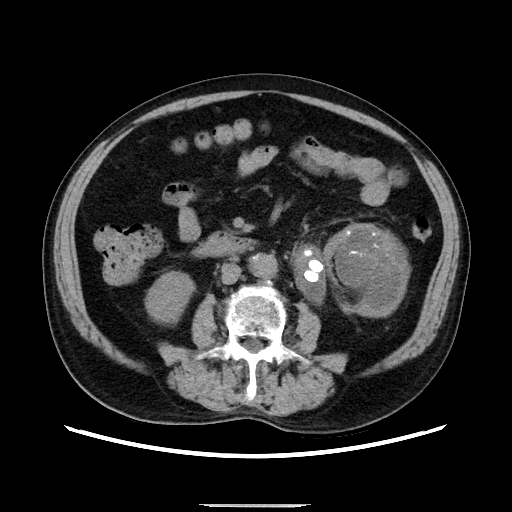

Supplement: Supplementary file 2 [file DataSheet2.zip › Figure S19.tif]

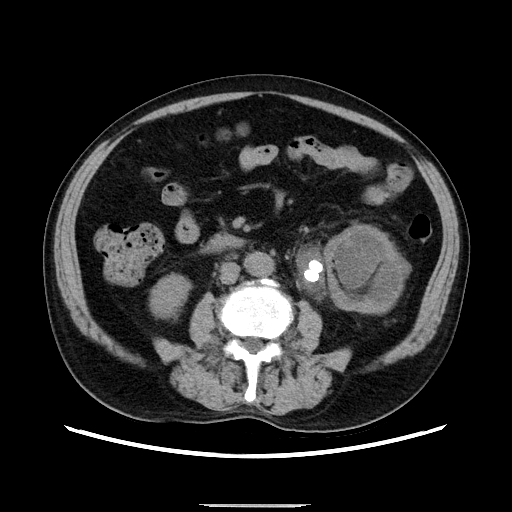

Supplement: Supplementary file 2 [file DataSheet2.zip › Figure S20.tif]

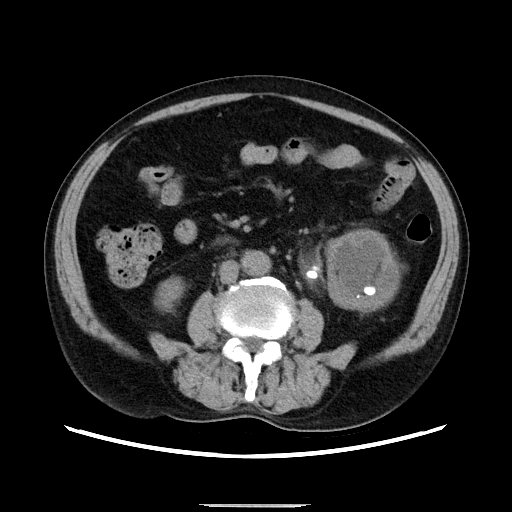

Supplement: Supplementary file 2 [file DataSheet2.zip › Figure S21.tif]

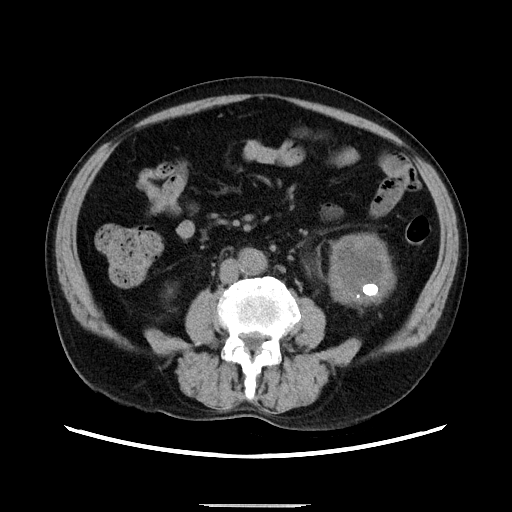

Supplement: Supplementary file 2 [file DataSheet2.zip › Figure S22.tif]

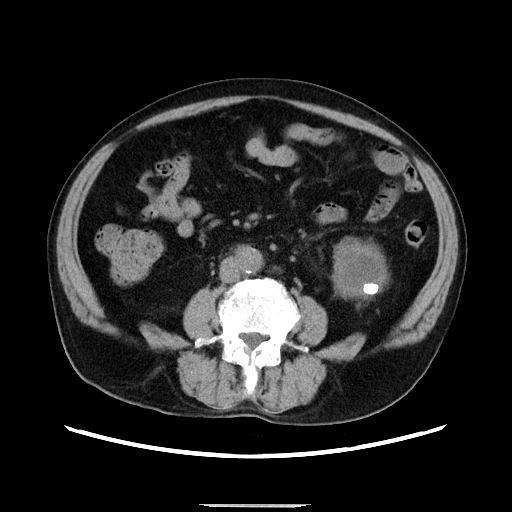

Supplement: Supplementary file 2 [file DataSheet2.zip › Figure S23.tif]

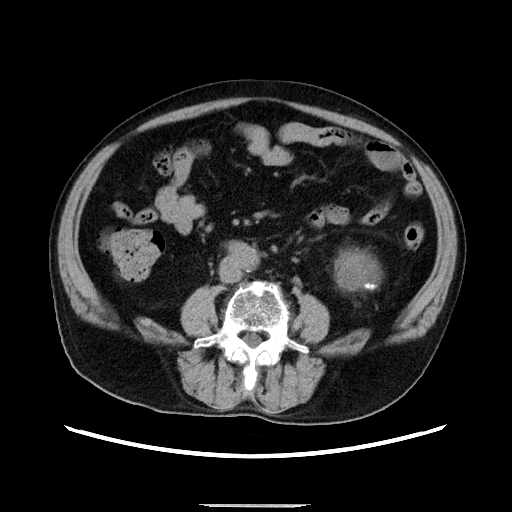

Supplement: Supplementary file 2 [file DataSheet2.zip › Figure S24.tif]

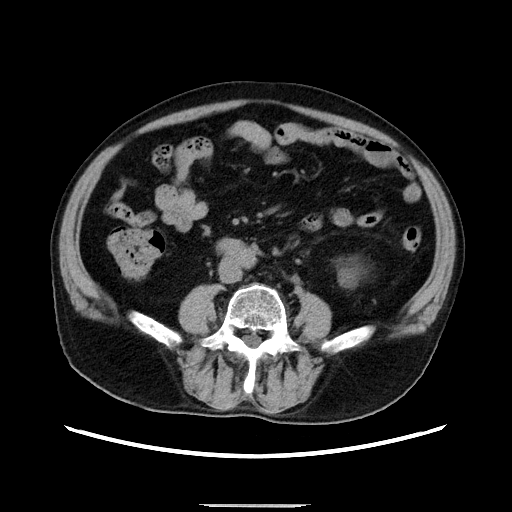

Supplement: Supplementary file 2 [file DataSheet2.zip › Figure S25.tif]

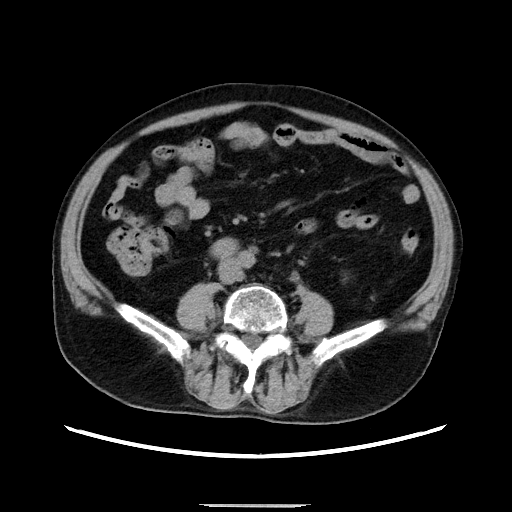

Supplement: Supplementary file 2 [file DataSheet2.zip › Figure S26.tif]
